# A transient third cranial nerve palsy as presenting sign of spontaneous intracranial hypotension

**DOI:** 10.1007/s10194-011-0345-1

**Published:** 2011-05-05

**Authors:** Antonio Russo, Alessandro Tessitore, Mario Cirillo, Alfonso Giordano, Rosa De Micco, Gennaro Bussone, Gioacchino Tedeschi

**Affiliations:** 1 Department of Neurological Sciences, Second University of Naples, 80138 Naples, Italy; 2 Institute for Diagnosis and Care “Hermitage Capodimonte”, 80131 Naples, Italy; 3 Pain Neuromodulation Unit, Department of Neurology, Headache Center, Carlo Besta Neurological Institute Foundation, 20133 Milan, Italy

**Keywords:** Intracranial hypotension, Third cranial nerve palsy, MRI, Epidural blood patch

## Abstract

Spontaneous intracranial hypotension is an uncommon cause of sudden and persistent headache: associated symptoms are common, among which there are cranial nerve palsies, especially of the abducens nerve. We report a case of a 21-year-old man with a transient and isolated third nerve palsy due to spontaneous intracranial hypotension. To our knowledge, there are only few reports in the literature of such association.

## Introduction

Spontaneous intracranial hypotension (SIH) is an uncommon cause of sudden and persistent headache and patients typically present with postural or exertional headaches that can be temporarily relieved by lying in a supine or recumbent position [1]. Associated symptoms are common, among which there are cranial nerve palsies [2], frequently resulting in ophthalmoplegia [3–8], especially of the abducens nerve [9]. Although rarely, third and fourth cranial nerves involvement have been previously reported [10]. Clinical symptoms are usually accompanied by magnetic resonance imaging (MRI) findings related to cerebrospinal fluid (CSF) depletion including subdural fluid collections, enhancement of the pachymeninges, engorgement of venous structures, pituitary hyperemia and sagging of the brain [11]. The present case, reporting the association with an isolated third nerve palsy, shows that a deep knowledge of SIH clinical presentation may avoid misdiagnoses.

## Case report

We report a case of a 21-year-old man, admitted to our hospital, for a sudden onset of a severe holocranial pain and diplopia. The headache was continuous, and changing position, particularly orthostatism, caused worsening of symptoms which conversely were relieved by lying flat. The patient did not complain about phonophobia, vertigo or tinnitus and vomiting, but reported photophobia and nausea.

Neurological examination was normal other than for cranial nerves evaluation that showed diplopia due to mild restricted adduction of the left eye toward right and ipsilateral partial ptosis. Pupils were equal and reactive to light (both directly and consensually) and accommodation. Diplopia disappeared within 2 h. Saccadic eye movements and ocular examination were normal with visual fields full to confrontation.

The patient did not experience head or neck trauma and fever or rashes were not present. To exclude a possible subarachnoid hemorrhage (SAH), the patient underwent a brain CT scan which was negative for hemorrhage. To rule out a diffuse inflammatory disease or neoplastic processes, routine blood, rheumatic and autoimmune tests, paraneoplastic markers, serum levels of angiotensin-converting enzyme, thoracic radiography and abdominal ultrasound, were performed and resulted normal.

**Fig. 1:**
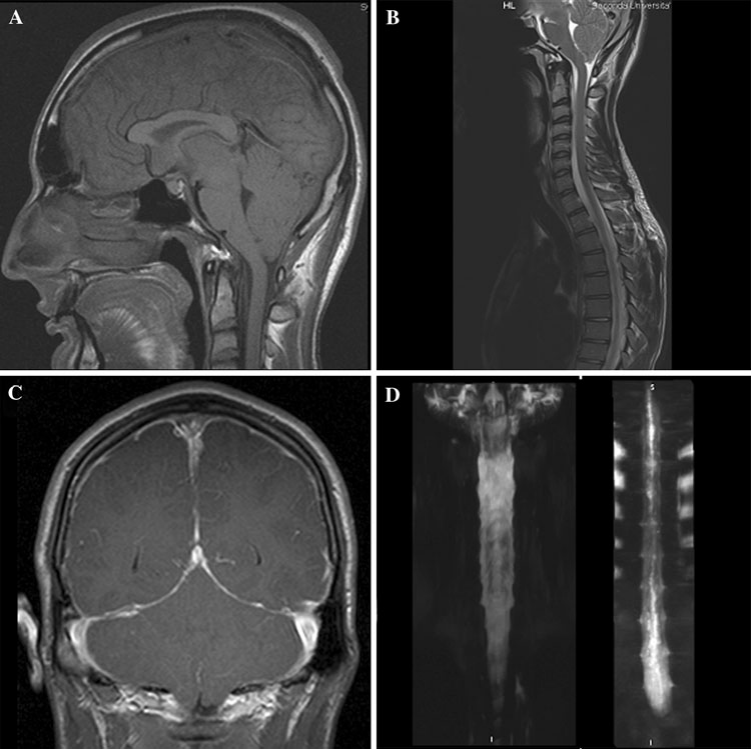
**a** Sagittal T1-weighted brain MRI, **b** sagittal T2-weighted spinal MRI, **c** coronal T1-weighted brain MRI post-gadolinium contrast administration, **d** MR myelogram of upper and lower spinal subdural space

Brain and spine magnetic resonance imaging (MRI) without contrast revealed a mild descent of the brainstem with mild cerebellar tonsillar herniation and flattening of pontine surface, dilated sagittal sinus and enlargement of the pituitary gland (Fig. 1a), associated with a subdural hematoma, surrounding bilaterally the fronto-parietal and temporo-polar regions. The latter findings were consistent with hygroma. Another subdural fluid collection was present in the anterior section of the spinal cord (at C_5_–D_1_ level) with a consequent posterior dislocation. In addition, symmetrically dilated vascular structures, with abnormal epidural venous engorgement, were again seen anterior to the cervical cord without cord compression, from C_4_ level up to atlanto**-**occipital junction (Fig. 1b). After gadolinium, MRI showed diffuse pachymeningeal enhancement in supratentorial and infratentorial regions (Fig. 1c). Dural enhancement was also notable in the spinal cord at C1–D1 level.

MR myelography showed no signs of spreading cerebrospinal fluid (CSF) at the level of the cervical and dorsal root pockets (Fig. 1d).

According to the International Headache Society (IHS) ICHD-II criteria, the MRI findings and the clinical history were consistent with a diagnosis of headache attributed to low CSF pressure. Although ICHD-II comments suggest that “dural puncture should be avoided in patients with positive MRI signs, such as meningeal enhancement with contrast” we performed CSF analysis to corroborate our diagnostic hypothesis. CSF opening pressure was of 5 cmH_2_O and a lymphocytic pleocytosis (20 cells/ml) was detected.

Initially, the case was managed by bed rest, hydration and steroidal therapy, but after 3 weeks of conservative treatment with limited benefit on the orthostatic headache, the SIH syndrome was successfully managed with the application of lumbar epidural blood patch (EBP) using approximately 28 mL of autologous blood, resulting in immediate relief of clinical symptoms. After 6 months, clinical follow-up revealed complete resolution of symptoms. In contrast, MRI findings were substantially unmodified, showing only a mild reduction of brain sagging, with persistent subdural fluid collection in the same regions, epidural venous engorgement and diffuse meningeal enhancement in supratentorial and infratentorial regions (images not shown).

## Discussion

To our knowledge, this is the first report of a transient and isolated third nerve palsy as the presenting sign of SIH. CSF exerts a necessary buoyant force on the cranial contents [2], suspending the brain and cranial nerves and protecting them from downward traction [12]. When the CSF volume decreases, the burden on the vascular and/or dural pain sensitive structures, subjected to traction and distortion, may cause the headache.

A relatively low CSF volume may result by either post-traumatic or spontaneous dural laceration. The first condition is most commonly secondary to iatrogenic causes. The second one can be frequently due to micro ruptures of the dura occurring at weak points along the spinal root sleeves (especially in the presence of connective tissue disorder) resulting in a syndrome known as SIH. However, CSF leakage cannot be demonstrated in several cases of SIH.

Orthostatic, diffuse or dull headache that worsens within 15 min after sitting or standing and that can be temporarily relieved by lying in a supine or recumbent position, is the main manifestation of SIH. Other common signs and symptoms include tinnitus, hypoacusia, vertigo, photophobia, nausea and neck stiffness. A variety of cranial nerve palsies is frequently associated with SIH (30–35%) [2] resulting in ophthalmoplegia and visual disturbances [6]. Abducens nerve palsy is the most common occurring in about 80% of reported patients with SIH-related ophthalmoplegia [2]. Although less common, both unilateral and bilateral paresis of the third and fourth cranial nerves might occur with SIH. Ferrante et al. [7] reported a woman with a paresis of the right third combined to sixth cranial nerves. Moreover, Warner [9] described an isolated, partial third cranial nerve palsy due to SIH, although eighth cranial nerve involvement was present in the form of “intermittent whistle” in the ear. More recently, Alonso-Canovas et al. [13] reported an incomplete left third cranial nerve palsy in the course of SIH.

Pathophysiology of cranial nerve paresis in SIH is not completely understood and potential causes include traction on cranial nerves due to downward displacement of cerebral structures and cranial nerve compression [1] or might be due to secondary brainstem compression and transitory ischemia in the acute phase of SIH.

## Conclusion

We believe that a deep knowledge of clinical presentation of SIH may avoid misdiagnoses, especially with SAH [2]. Indeed, the presence of third cranial nerve palsy (alone or in association with other cranial nerves involvement) with sudden onset orthostatic headache should prompt a possible diagnosis of SIH which must be considered only after SAH has been carefully ruled out. Usually after EBP procedure, clinical symptoms recede together with the disappearance of MRI features, however, in line with the present case, a clinical–radiological dissociation in which clinical syndrome improved, while MRI features did not, has been already reported [5].
